# Characterizing the hypertensive cardiovascular phenotype in the UK Biobank

**DOI:** 10.1093/ehjci/jead123

**Published:** 2023-06-13

**Authors:** Hussein Elghazaly, Celeste McCracken, Liliana Szabo, James Malcolmson, Charlotte H Manisty, Alun H Davies, Stefan K Piechnik, Nicholas C Harvey, Stefan Neubauer, Saidi A Mohiddin, Steffen E Petersen, Zahra Raisi-Estabragh

**Affiliations:** Department of Surgery and Cancer, Imperial College London and Imperial College NHS Trust, South Kensington, SW7 2BX London, UK; Division of Cardiovascular Medicine, Radcliffe Department of Medicine, University of Oxford, National Institute for Health Research Oxford Biomedical Research Centre, Oxford University Hospitals NHS Foundation Trust, Oxford OX3 9DU, UK; William Harvey Research Institute, NIHR Barts Biomedical Research Centre, Queen Mary University of London, Charterhouse Square, London EC1M 6BQ, UK; Barts Heart Centre, St Bartholomew’s Hospital, Barts Health NHS Trust, West Smithfield, London EC1A 7BE, UK; Semmelweis University, Heart and Vascular Center, BudapestHungary; Barts Heart Centre, St Bartholomew’s Hospital, Barts Health NHS Trust, West Smithfield, London EC1A 7BE, UK; Barts Heart Centre, St Bartholomew’s Hospital, Barts Health NHS Trust, West Smithfield, London EC1A 7BE, UK; Institute of Cardiovascular Science, University College London, London, UK; Department of Surgery and Cancer, Imperial College London and Imperial College NHS Trust, South Kensington, SW7 2BX London, UK; Division of Cardiovascular Medicine, Radcliffe Department of Medicine, University of Oxford, National Institute for Health Research Oxford Biomedical Research Centre, Oxford University Hospitals NHS Foundation Trust, Oxford OX3 9DU, UK; MRC Lifecourse Epidemiology Centre, University of Southampton, Southampton SO16 6YD, UK; NIHR Southampton Biomedical Research Centre, University of Southampton and University Hospital Southampton NHS Foundation Trust, Southampton SO16 6YD, UK; Division of Cardiovascular Medicine, Radcliffe Department of Medicine, University of Oxford, National Institute for Health Research Oxford Biomedical Research Centre, Oxford University Hospitals NHS Foundation Trust, Oxford OX3 9DU, UK; William Harvey Research Institute, NIHR Barts Biomedical Research Centre, Queen Mary University of London, Charterhouse Square, London EC1M 6BQ, UK; Barts Heart Centre, St Bartholomew’s Hospital, Barts Health NHS Trust, West Smithfield, London EC1A 7BE, UK; William Harvey Research Institute, NIHR Barts Biomedical Research Centre, Queen Mary University of London, Charterhouse Square, London EC1M 6BQ, UK; Barts Heart Centre, St Bartholomew’s Hospital, Barts Health NHS Trust, West Smithfield, London EC1A 7BE, UK; Health Data Research UK, London, UK; Alan Turing Institute, London, UK; William Harvey Research Institute, NIHR Barts Biomedical Research Centre, Queen Mary University of London, Charterhouse Square, London EC1M 6BQ, UK; Barts Heart Centre, St Bartholomew’s Hospital, Barts Health NHS Trust, West Smithfield, London EC1A 7BE, UK

**Keywords:** cardiovascular magnetic resonance, ethnicity, women’s health, population health, antihypertensive therapies

## Abstract

**Aims:**

To describe hypertension-related cardiovascular magnetic resonance (CMR) phenotypes in the UK Biobank considering variations across patient populations.

**Methods and results:**

We studied 39 095 (51.5% women, mean age: 63.9 ± 7.7 years, 38.6% hypertensive) participants with CMR data available. Hypertension status was ascertained through health record linkage. Associations between hypertension and CMR metrics were estimated using multivariable linear regression adjusting for major vascular risk factors. Stratified analyses were performed by sex, ethnicity, time since hypertension diagnosis, and blood pressure (BP) control. Results are standardized beta coefficients, 95% confidence intervals, and *P*-values corrected for multiple testing. Hypertension was associated with concentric left ventricular (LV) hypertrophy (increased LV mass, wall thickness, concentricity index), poorer LV function (lower global function index, worse global longitudinal strain), larger left atrial (LA) volumes, lower LA ejection fraction, and lower aortic distensibility. Hypertension was linked to significantly lower myocardial native T1 and increased LV ejection fraction. Women had greater hypertension-related reduction in aortic compliance than men. The degree of hypertension-related LV hypertrophy was greatest in Black ethnicities. Increasing time since diagnosis of hypertension was linked to adverse remodelling. Hypertension-related remodelling was substantially attenuated in hypertensives with good BP control.

**Conclusion:**

Hypertension was associated with concentric LV hypertrophy, reduced LV function, dilated poorer functioning LA, and reduced aortic compliance. Whilst the overall pattern of remodelling was consistent across populations, women had greater hypertension-related reduction in aortic compliance and Black ethnicities showed the greatest LV mass increase. Importantly, adverse cardiovascular remodelling was markedly attenuated in hypertensives with good BP control.

## Graphical Abstract

**Graphical Abstract:**
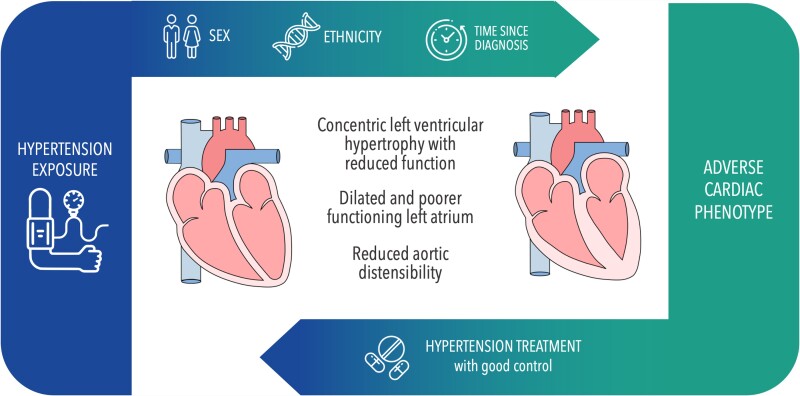

**See the editorial comment for this article ‘Modelling relations between blood pressure, cardiovascular phenotype, and clinical factors using large scale imaging data’, by T. Kart *et al*., https://doi.org/10.1093/ehjci/jead161.**

## Introduction

Hypertension is a major cardiovascular risk factor, with an estimated global prevalence of over one billion people.^1^ Hypertensive heart disease comprises a constellation of adverse phenotypic alterations of cardiac structure and function.^2^ Whilst several imaging techniques are available to characterise the hypertensive heart, cardiovascular magnetic resonance (CMR) provides superior reproducibility for quantification of cardiac chamber volumes, mass, and function as well as uniquely allowing characterization of myocardial fibrosis.^3^

Previous studies have investigated hypertension-related cardiac remodelling using CMR in select clinical samples.^2,4–6^ However, this subject has not been adequately studied in large population-based cohorts. Furthermore, while sex and ethnicity differences in the burden, treatment response, and health consequences of hypertension are widely recognized,^7,8^ there is little data describing variations in cardiac remodelling associated with these demographic factors. Characterizing hypertension-related cardiovascular remodelling and heterogeneities in different populations is essential for understanding the mechanisms through which hypertension leads to disease and variation in risk across patient groups.

We characterised the hypertensive cardiovascular phenotype in ∼40 000 community-dwelling participants from the UK Biobank, considering variations by sex, ethnicity, duration of exposure, and blood pressure (BP) control.

## Methods

### UK Biobank, study population and setting

The UK Biobank is a large prospective cohort of over 500 000 participants.^9^ UK residents aged 40–69 years old, were identified through National Health Service registers and recruited between 2006 and 2010^9^. Baseline assessment comprised detailed characterization of socio-demographics, lifestyle factors, medical history, and a series of physical measures.^9^ The UK Biobank Imaging Study (2015-ongoing), which includes CMR, aims to scan 100 000 of the original participants.^10^ Many of the baseline assessments are repeated at the imaging visit. Extensive health record linkages have been established for the UK Biobank cohort.

### Hypertension status

We considered two approaches to defining hypertension. Firstly, we considered clinically diagnosed hypertension (binary) ascertained based on record of a hypertension diagnosis at time of imaging in any of the linked databases or UK Biobank assessment questions (see Supplementary data online, *Table S1*). Second, in supplementary analyses, we considered systolic BP (SBP) as the exposure of interest (continuous variable). Participants had two BP measurements at the time of imaging. We defined SBP using the average of the two readings, limiting to values between 60 to 220 mmHg. Duration of hypertension exposure was defined by the length of time between first recorded clinical diagnosis of hypertension and the imaging visit, categorized as ≤5 years, 6–10 years, 11–20 years, or >20 years.

### Hypertension treatment status

To examine variations by BP control, we limited the sample to clinically diagnosed hypertensives with antihypertensive medications listed in primary care prescription records (i.e. ‘treated hypertension’). As per the National Institute for Health and Care Excellence (NICE) guidelines,^11^ we considered the following medication classes as anti-hypertensives: angiotensin converting enzyme inhibitors, angiotensin receptor blockers, calcium channel blockers, thiazide diuretics, alpha-blockers, beta-blockers, and mineralocorticoid receptor antagonists (see Supplementary data online, *Table S2*). We stratified this subset of participants according to whether their SBP recorded at imaging was above or below the age-specific target BP recommended by NICE.^11^ Participants were assigned the label of ‘good control’ where SBP was <140 mmHg for adults aged under 80 and SBP <150 mmHg for adults aged 80 and over, or ‘poor control’ where SBP was above these target thresholds.

### CMR image acquisition and analysis

CMR scans were performed using 1.5 T scanners (MAGNETOM Aera, Synge Platform VD13A, Siemens Healthcare, Erlangen, Germany).^10^ Fully automated image analysis pipelines with in-built quality control were trained and validated on a large ground truth manual analysis dataset of over 5000 UK Biobank studies.^12–14^ Participants without a valid (unavailable or poor quality) CMR metric were excluded from analysis for that metric. We included the following measures: Left atrial volume index (LAVi), left atrial ejection fraction (LAEF), Left ventricular end diastolic volume index, left ventricular mass index (LVMi), left ventricular mass-to-end diastolic volume ratio (LVM/LVEDV), maximal wall thickness (MWT), left ventricular ejection fraction (LVEF), left ventricular systolic volume index (LVSVi), left ventricular global function index (LVGFI), global longitudinal strain (GLS), myocardial Native T1 (global mid-ventricular short axis slice), aortic distensibility (AoD).

As per previous reports,^15^ LVGFI (%) was calculated as LV stroke volume/LV global volume × 100, where LV global volume was calculated as the sum of the LV mean cavity volume [(LV end-diastolic volume + LV end-systolic volume)/2] and myocardium volume (LV mass/density). Density of LV was specified as 1.05 g/mL. Aortic distensibility is a direct measure of local arterial stiffness determined by the change in aortic cross-sectional area in systole-diastole (i.e. aortic strain) divided by central pulse pressure (in mmHg).^16^

### Ascertainment of covariates

Participant age was recorded at the imaging visit. Sex and ethnicity were self-reported. Ethnicity categories supplied by the UK Biobank are: ‘White’, ‘Black or Black British’, ‘Asian or Asian British’, ‘Chinese’, ‘Mixed’, and ‘Other’. In the UK Biobank, ‘Asian or Asian British’ refers to Indian, Pakistani, Bangladeshi, or ‘any other Asian’ background. Smoking status and alcohol intake were self-reported. The Townsend deprivation index was calculated immediately prior to participants joining the UK Biobank based on the preceding national census output areas. The score incorporates four elements of employment, car ownership, home ownership, and household overcrowding.^17^ Each participant is assigned a score corresponding to the output area in which their postcode is located. A score of zero indicates deprivation equivalent to national averages, whilst positive scores indicate greater, and negative values lower, deprivation levels. Body mass index (BMI) was calculated from height and weight measured at imaging. Diabetes and high cholesterol status, at time of imaging, were defined from record of the diagnosis in any of the linked databases or UK Biobank assessment questions (see Supplementary data online, *Table S1*).

### Statistical analysis

Analysis was performed using R version 4.1.2 and RStudio 2022.07.1. Descriptive statistics are presented as mean (standard deviation) or median [25th percentile, 75th percentile] depending on distribution skewness. Associations between diagnosed hypertension and each CMR metric were assessed using multivariable linear regression, adjusted for age, sex, ethnicity, Townsend index, alcohol intake frequency, BMI, smoking, diabetes, and high cholesterol. We performed stratified analyses by sex, ethnicity, and time since diagnosis, and among treated hypertensives, by BP control (good vs. poor control). In sensitivity analyses, associations were re-examined with SBP set as the exposure of interest.

Associations are reported as standardized beta coefficients with corresponding 95% confidence intervals (CIs) and *P*-values. Significance thresholds were adjusted for multiple testing using the Benjamini–Hochberg method^18^ with a 5% false discovery rate.

## Results

### Baseline characteristics

The study includes 39 095 participants (see Supplementary data online, *Figure S1*). The mean age was 63.9 ± 7.7 years, 48.5% of the participants were male, and 97.0% were from White ethnic backgrounds (*Table 1*). We identified 15 107 participants with a clinical diagnosis of hypertension. These individuals had greater burden of cardiovascular risk factors. The prevalence of diabetes and high cholesterol in the hypertensive group were 11.2% and 51.0%, compared to 2.5% and 15.8% in the non-hypertensive cohort, respectively. Amongst those with hypertension, 80.2% (12 119/15 107) had a record of antihypertensive medications, with 37.0% (5594/15 107) on three or more different antihypertensive classes (*Table 1*). Averages for CMR phenotypes stratified by hypertension status are presented in Supplementary data online, *Table S3*.

**Table 1:** Sample characteristics

	Whole sample (*n* = 39 095)	Diagnosed Hypertension (*n* = 15 107, 38.6%)	No Hypertension (*n* = 23 988, 61.4%)
Age (years)	63.9 (±7.7)	66.1 (±7.3)	62.6 (±7.6)
Women	20 138 (51.5%)	6574 (43.5%)	13 564 (56.5%)
Men	18 957 (48.5%)	8533 (56.5%)	10 424 (43.5%)
Ethnicity group			
White	37 903 (97.0%)	14 629 (96.8%)	23 274 (97.0%)
Asian	410 (1.0%)	174 (1.2%)	236 (1.0%)
Black	246 (0.6%)	122 (0.8%)	124 (0.5%)
Chinese	109 (0.3%)	30 (0.2%)	79 (0.3%)
Mixed	180 (0.5%)	61 (0.4%)	119 (0.5%)
Other	170 (0.4%)	61 (0.4%)	109 (0.5%)
(Missing)	77 (0.2%)	30 (0.2%)	47 (0.2%)
Smoking status			
Never smoked	24 343 (62.3%)	8728 (57.8%)	15 615 (65.1%)
Previous smoker	13 368 (34.2%)	5875 (38.9%)	7493 (31.2%)
Current smoker	1384 (3.5%)	504 (3.3%)	880 (3.7%)
Alcohol intake frequency			
Never	2525 (6.5%)	1069 (7.1%)	1456 (6.1%)
Less than once per week	8428 (21.6%)	3346 (22.1%)	5082 (21.2%)
Once weekly or more	27 926 (71.4%)	10 593 (70.1%)	17 333 (72.3%)
(Missing)	216 (0.6%)	99 (0.7%)	117 (0.5%)
Body mass index (kg/m^2^)	25.9 [23.5, 28.8]	27.1 [24.6, 30.2]	25.2 [23.0, 27.8]
Townsend deprivation score	−2.6 [−3.9, −0.6]	−2.6 [−3.9, −0.5]	−2.7 [−3.9, −0.6]
Diabetes	2286 (5.8%)	1692 (11.2%)	594 (2.5%)
High cholesterol	11 488 (29.4%)	7706 (51.0%)	3782 (15.8%)
SBP at imaging (mmHg)	138.9 (±18.7)	145.3 (±18.7)	134.9 (±17.5)
SBP category			
At or above 140 mmHg (note)	17 606 (45.0%)	9014 (59.7%)	8592 (35.8%)
Below 140 mmHg	21 489 (55.0%)	6093 (40.3%)	15 396 (64.2%)
Duration of hypertension			
Five years or less	—	2642 (17.5%)	—
Six to 10 years	—	2653 (17.6%)	—
Eleven to 20 years	—	5849 (38.7%)	—
More than 20 years	—	3963 (26.2%)	—
Antihypertensive medications			
None	—	2988 (19.8%)	—
One or two	—	6525 (43.2%)	—
Three or more	—	5594 (37.0%)	—

Counts variables are presented as number (percentage), continuous variables as mean (standard deviation) or median (inter-quartile range).

SBP, systolic blood pressure.

### Association of hypertension with cardiovascular phenotypes

**Figure 1:**
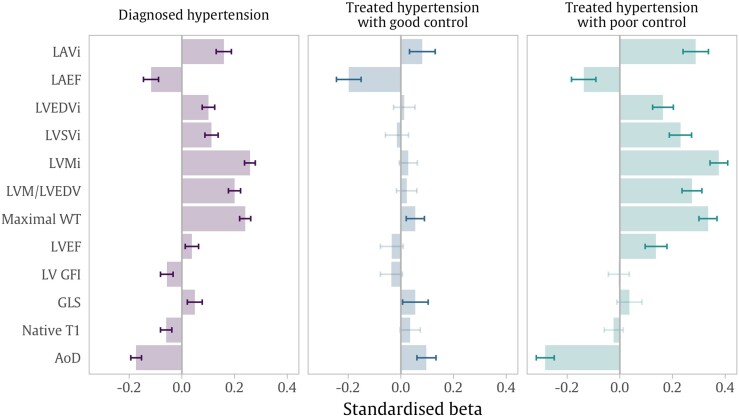
Results are standardised beta coefficients representing standard deviation change in CMR metrics associated with diagnosed hypertension status in the whole sample in the first column. The second and third columns indicate the difference observed in treated hypertension groups as confirmed by primary care records, divided by good vs. poor blood pressure control. For all models, the reference group is no hypertension. Models include adjustment for age, sex, ethnicity, deprivation, alcohol, body mass index, smoking, diabetes, and high cholesterol. Darker coloured notches indicate significant *P*-values after multiple testing adjustment. CMR: cardiovascular magnetic resonance, i: indexed to body surface area, LAVi: maximum left atrial volume, LAEF: left atrial ejection fraction, LVEDVi: left ventricular end-diastolic volume, LVSVi: left ventricular stroke volume, LVMi: left ventricular mass, LVM/LVEDV: left ventricular mass to volume ratio, WT: wall thickness, LVEF: left ventricular ejection fraction, LVGFI: left ventricular global function index, GLS: Global Longitudinal Strain, AoD: aortic distensibility, SBP: systolic blood pressure.

In fully adjusted linear regression models, hypertension was associated with larger LAVi and lower LAEF (*Figure 1*, *Table 2*). The largest magnitude of positive association was seen with LVMi (Beta = 0.258, 95% CI = [0.238, 0.279]), MWT (Beta = 0.240, 95% CI = [0.219, 0.261]), and LVM/LVEDV (Beta = 0.200, 95% CI = [0.177, 0.223]). Hypertension was associated with higher LVEF and LVSVi. At the same time, we found significant association of hypertension with lower LVGFI and worse GLS values. Hypertension was associated with lower AoD There was a negative association between hypertension and native T1. These associations were consistent in models with SBP set as the exposure of interest (see Supplementary data online, *Table S4*).

**Table 2:** Associations of hypertension with CMR metrics in fully adjusted linear regression models stratified by sex

	Whole sample	Women	Men	Hypertension × sex interaction
LAVi	0.159* [0.130, 0.188]	0.142* [0.105, 0.180]	0.176* [0.132, 0.220]	0.050 [−0.002, 0.103]
	3.81 × 10^−27^	1.60 × 10^−13^	5.26 × 10^−15^	0.0617
LAEF	−0.117* [−0.146, −0.089]	−0.124* [−0.162, −0.086]	−0.108* [−0.151, −0.065]	−0.017 [−0.069, 0.035]
	1.08 × 10^−15^	1.52 × 10^−10^	9.77 × 10^−7^	0.5218
LVEDVi	0.101* [0.077, 0.125]	0.088* [0.060, 0.116]	0.119* [0.080, 0.157]	−0.020 [−0.063, 0.024]
	8.31 × 10^−17^	1.36 × 10^−9^	1.29 × 10^−9^	0.3741
LVSVi	0.113* [0.088, 0.138]	0.109* [0.077, 0.141]	0.121* [0.082, 0.160]	−0.037 [−0.082, 0.009]
	1.42 × 10^−18^	1.61 × 10^−11^	1.42 × 10^−9^	0.1149
LVMi	0.258* [0.238, 0.279]	0.238* [0.213, 0.262]	0.283* [0.250, 0.316]	0.005 [−0.032, 0.042]
	2.20 × 10^−133^	2.70 × 10^−80^	2.74 × 10^−62^	0.7873
LVM/LVEDV	0.200* [0.177, 0.223]	0.206* [0.177, 0.235]	0.193* [0.157, 0.228]	−0.003 [−0.045, 0.039]
	2.21 × 10^−64^	5.16 × 10^−44^	7.70 × 10^−26^	0.8788
Maximal WT	0.240* [0.219, 0.261]	0.243* [0.217, 0.269]	0.236* [0.203, 0.269]	−0.001 [−0.039, 0.038]
	8.51 × 10^−110^	2.40 × 10^−73^	1.37 × 10^−44^	0.9759
LVEF	0.038* [0.013, 0.063]	0.048* [0.014, 0.082]	0.029 [−0.008, 0.067]	−0.030 [−0.076, 0.017]
	0.0032	0.0057	0.1274	0.2097
LV GFI	−0.057* [−0.081, −0.033]	−0.058* [−0.092, −0.024]	−0.056* [−0.090, −0.023]	0.003 [−0.040, 0.047]
	2.66 × 10^−6^	7.61 × 10^−4^	0.0011	0.8881
GLS	0.049* [0.021, 0.077]	0.042 [0.002, 0.082]	0.055* [0.015, 0.095]	0.025 [−0.027, 0.076]
	6.91 × 10^−4^	0.0394	0.0069	0.3475
Native T1	−0.060* [−0.081, −0.038]	−0.117* [−0.148, −0.087]	−0.008 [−0.038, 0.023]	0.210* [0.170, 0.249]
	5.91 × 10^−8^	4.07 × 10^−14^	0.6243	1.17 × 10^−25^
AoD	−0.174* [−0.194, −0.153]	−0.199* [−0.228, −0.169]	−0.146* [−0.174, −0.119]	0.076* [0.039, 0.113]
	2.38 × 10^−62^	4.54 × 10^−39^	2.39 × 10^−25^	6.46 × 10^−5^

Results are standardised Beta coefficients, 95% confidence intervals, and *P*-values, representing standard deviation change in CMR metrics associated with hypertension (vs. no hypertension). Models include adjustment for age, sex, ethnicity, deprivation, alcohol, body mass index, smoking, diabetes, and high cholesterol.

CMR, cardiovascular magnetic resonance; i, indexed to body surface area; LAVi, maximum left atrial volume; LAEF, left atrial ejection fraction; LVEDVi, left ventricular end-diastolic volume; LVSVi, left ventricular stroke volume; LVMi, left ventricular mass; LVM/LVEDV, left ventricular mass to volume ratio; WT, wall thickness; LVEF, left ventricular ejection fraction; LVGFI, left ventricular global function index; GLS, Global Longitudinal Strain; AoD, aortic distensibility.

significant *P*-value after multiple testing adjustment.

### Sex differential remodelling

We observed an overall similar pattern of hypertension-related cardiovascular remodelling in men and women across all CMR metrics (*Table 2*). There was evidence of significant sex interaction for associations with AoD and myocardial native T1. In fully adjusted sex stratified analyses, hypertension was associated with lower AoD in both men (Beta = -0.146, [-0.174, -0.119], *P* = 2.39 × 10^−25^) and women (Beta = -0.199, 95% CI = [-0.228, -0.169], *P* = 4.54 × 10^−39^) but with greater magnitude of effect in women. The association of hypertension with lower native T1 in the whole cohort was driven by relationships in women (Beta = -0.117, 95% CI = [-0.148, -0.087], *P* = 4.07 × 10^−14^) and attenuated to the null in men (Beta = -0.008, 95% CI = [-0.038, 0.023], *P* = 0.62). We observed similar results in models with SBP set as the exposure of interest (see Supplementary data online, *Table S4*).

### Time since diagnosis

**Figure 2:**
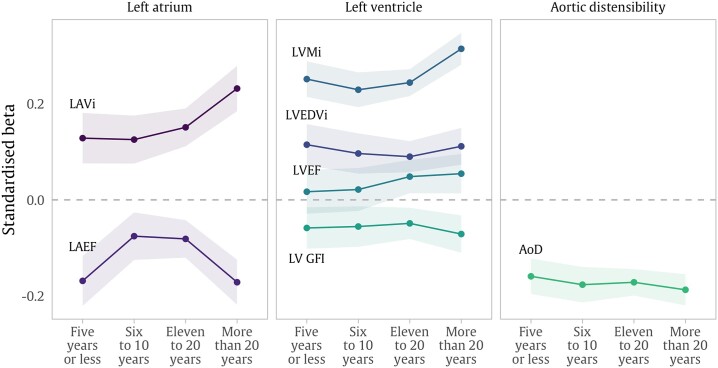
Selected associations between hypertension and CMR metrics in fully adjusted models stratified by time since hypertension diagnosis. Points are standardised beta coefficients representing SD change in CMR metrics associated with hypertension status in participants stratified by the period of time since hypertension diagnosis. The zero horizontal line represents average CMR in participants with no hypertension. Shaded areas represent the 95% confidence limits for each coefficient. Models include adjustment for age, sex, ethnicity, deprivation, alcohol, body mass index, smoking, diabetes, and high cholesterol. CMR: cardiovascular magnetic resonance, i: indexed to body surface area, LAVi: maximum left atrial volume, LAEF: left atrial ejection fraction, LVEDVi: left ventricular end-diastolic volume, LVSVi: left ventricular stroke volume, LVMi: left ventricular mass, LVM/LVEDV: left ventricular mass to volume ratio, WT: wall thickness, LVEF: left ventricular ejection fraction, LVGFI: left ventricular global function index, GLS: Global Longitudinal Strain, AoD: aortic distensibility.

Independent of all other covariates, participants with greater time since diagnosis of hypertension, had larger magnitude of effect in positive associations with LVMi, MWT, and LVM/LVEDV (*Figure 2*, Supplementary data online, *Table S5*). Hypertension was not significantly associated with LVEF among participants diagnosed with hypertension for <11 years. However, in those with longer time since diagnosis (≥11 years), hypertension was associated with significantly increased LVEF. Participants with greater time since diagnosis had larger magnitude of association between hypertension and lower AoD. Increasing time since diagnosis of hypertension was associated with progressively larger degree of positive association with LAVi, but with a much steeper increase in effect size for those with the longest duration of exposure (>20 years). For LAEF, the magnitude of reduction was greatest in participants who were diagnosed with hypertension for ≤5 years or ≥20 years.

### BP control

Compared to non-hypertensives, individuals with treated hypertension and poor BP control had similar remodelling patterns as described in the entire hypertensive cohort, but with larger effect sizes (*Table 3*, *Figure 1*). In participants with treated hypertension and good BP control, many of the hypertension-CMR associations were attenuated to the null. The only indicators of significant LV remodelling in this subset were higher MWT and poorer GLS. Surprisingly, those with well-controlled treated hypertension had slightly higher AoD than the group without hypertension diagnosis. Observed LAVi enlargement was noticeably greater in those with poor BP control vs. good BP control (Beta = 0.288 vs. 0.082), whilst the reduction in LAEF was greater in those with good control. These relationships were broadly consistent between men and women (see Supplementary data online, *Table S6*).

**Table 3:** Associations between CMR metrics treated hypertensives stratified into good and poor control, all compared with no hypertension

Metric	Effect of treated hypertension compared to no hypertension		
	Whole set (*n* = 6423)	Good control (*n* = 2826)	Poor control (*n* = 3597)
LAVi	0.176* [0.139, 0.214]	0.082* [0.033, 0.130]	0.288* [0.240, 0.336]
	4.25 × 10^−20^	0.0010	1.57 × 10^−31^
LAEF	−0.155* [−0.192, −0.119]	−0.198* [−0.245, −0.151]	−0.137* [−0.184, −0.091]
	9.32 × 10^−17^	1.38 × 10^−16^	6.55 × 10^−9^
LVEDVi	0.084* [0.053, 0.115]	0.013 [−0.028, 0.054]	0.164* [0.124, 0.203]
	9.09 × 10^−8^	0.5307	3.82 × 10^−16^
LVSVi	0.108* [0.075, 0.141]	−0.015 [−0.059, 0.029]	0.230* [0.188, 0.272]
	1.33 × 10^−10^	0.5080	1.33 × 10^−26^
LVMi	0.210* [0.184, 0.237]	0.028 [−0.006, 0.063]	0.376* [0.342, 0.410]
	6.16 × 10^−55^	0.1050	1.81 × 10^−105^
LVM/LVEDV	0.162* [0.132, 0.191]	0.022 [−0.017, 0.061]	0.274* [0.236, 0.311]
	1.54 × 10^−26^	0.2659	1.45 × 10^−45^
Maximal WT	0.200* [0.173, 0.227]	0.055* [0.021, 0.090]	0.335* [0.300, 0.369]
	1.07 × 10^−47^	0.0018	1.73 × 10^−80^
LVEF	0.058* [0.026, 0.091]	−0.034 [−0.078, 0.009]	0.138* [0.096, 0.179]
	4.82 × 10–4	0.1183	7.69 × 10^−11^
LV GFI	−0.022 [−0.053, 0.010]	−0.036 [−0.078, 0.006]	−0.004 [−0.044, 0.036]
	0.1777	0.0894	0.8435
GLS	0.043* [0.006, 0.080]	0.055* [0.007, 0.104]	0.036 [−0.011, 0.084]
	0.0227	0.0258	0.1360
Native T1	−0.005 [−0.033, 0.024]	0.036 [−0.003, 0.074]	−0.023 [−0.059, 0.013]
	0.7444	0.0702	0.2038
AoD	−0.118* [−0.145, −0.091]	0.097* [0.061, 0.133]	−0.283* [−0.317, −0.249]
	1.46 × 10^−17^	1.42 × 10^−7^	2.72 × 10^−59^

Results are standardised Beta coefficients, 95% confidence intervals, and *P*-values, representing standard deviation change in CMR metrics associated with hypertension (vs. no hypertension). ‘Good control’ indicates SBP <140 mmHg for adults aged <80 years and SBP <150 mmHg for adults aged ≥80 years. ‘Poor control’ indicates SBP above these thresholds. The reference group in all cases is no hypertension (*n* = 23 988). Models include adjustment for age, sex, ethnicity, deprivation, alcohol, body mass index, smoking, diabetes, and high cholesterol.

CMR, cardiovascular magnetic resonance; i, indexed to body surface area; LAVi, maximum left atrial volume; LAEF, left atrial ejection fraction; LVEDVi, left ventricular end-diastolic volume; LVSVi, left ventricular stroke volume; LVMi, left ventricular mass; LVM/LVEDV, left ventricular mass to volume ratio; WT, wall thickness; LVEF, left ventricular ejection fraction; LVGFI, left ventricular global function index; GLS, Global Longitudinal Strain; AoD, aortic distensibility.

significant *P*-value after multiple testing adjustment.

### Ethnicity differential patterns

The direction and pattern of associations between hypertension status and CMR metrics were consistent across all ethnic groups (see Supplementary data online, *Table S7*). Association of hypertension with higher LVMi (Beta = 0.363 vs. 0.257 in White ethnicities) and poorer LV function by LVGFI (Beta = -0.345 vs. -0.055 in White ethnicities) were greatest in Black ethnicities than in any other ethnic group. Whilst hypertension was associated with lower AoD across all ethnicities, the magnitude of effect was greatest in Chinese and Asian or Asian British (Beta = -0.191 and -0.406, respectively, vs. -0.173 in White ethnicities) ethnic groups.

## Discussion

### Summary of findings

In this large population-based study of 39 095 UK Biobank participants, we demonstrate a distinct adverse cardiovascular phenotype associated with hypertension. The prominent remodelling pattern was that of concentric LV hypertrophy (higher mass, greater wall thickness) and poorer LV function by LVGFI and GLS. We also demonstrate associations of hypertension with a larger and poorer functioning left atrium, in keeping with elevated LV filling pressures and diastolic dysfunction. Hypertension was linked to higher LVEF, likely reflecting compensatory adaptations in the setting of hypertension and geometric assumptions in calculation of this metric. Whilst the phenotype of the hypertensive heart was broadly similar in both men and women, there was evidence of greater hypertension-related reduction in aortic compliance in women than men. Furthermore, our results shed light into the natural history of hypertension-related cardiac remodelling, demonstrating greater concentric LV hypertrophy and worsening diastolic dysfunction by LA metrics in subsets with greater time since diagnosis of hypertension. Importantly, we found notable attenuation of hypertension-CMR associations in treated hypertensives with good (vs. poor) BP control. Finally, we demonstrate ethnic variations in the degree of cardiovascular remodelling, noting greater LV hypertrophy in Asian and Black ethnicities and greater reduction in aortic compliance in Chinese ethnic groups.

### Comparison with existing literature

The most prominent cardiovascular remodelling pattern associated with hypertension in our study was that of concentric LV hypertrophy, which reflects an adaptive response to increased arterial afterload occurring as a consequence of systemic hypertension.^2^ Our observations are consistent with several previous reports and corroborate the findings of these existing works in a much larger cohort.

We observed a larger magnitude of increase in LVMi in response to hypertension in Black ethnicities than other ethnic groups. Asian and Asian British ethnicities also had more prominent hypertension-related hypertrophic LV remodelling than other ethnicities, second to Black ethnicities. Chinese ethnicities had the greatest reduction in aortic compliance related to hypertension than any other ethnic group. There is limited data on ethnic differences in hypertension-related cardiac remodelling. Consistent with our findings, in a study of 82 hypertensives (44 Black, 38 White), Mohamed et al. report greater propensity to LV hypertrophy in Black individuals.^19^ Our study is the first to report differential CMR remodelling patterns in other ethnic groups.

We found that hypertension was associated with higher LVEF. This phenomenon is previously described in the literature^20–22^ and likely reflects compensatory adaptations and geometric assumptions in calculation of this metric. Although little data is available on the clinical significance of this phenomenon, supranormal LVEF has been linked to a higher risk of adverse cardiovascular outcomes in hypertensive patients.^23^ We found that LVGFI, a novel measure of LV function which incorporates correction for LV structure, was significantly reduced in association with hypertension. Previous reports have identified LVGFI as a predictor of heart failure and cardiovascular events with incremental utility over LVEF.^25,15,24^ Our results demonstrate significant association between hypertension and lower LVGFI in a larger population-based cohort. We additionally observed significant association of hypertension with poorer GLS values, an emerging measure of longitudinal function loss.^2,26^ To our knowledge, this is the first study to demonstrate an association between hypertension and GLS in a large cohort using CMR. Thus, our findings significantly extend existing literature in demonstrating associations of hypertension with two novel metrics: LVGFI and GLS, and in illustrating paradoxical hypertension–LVEF relationships.

We observed significant associations of hypertension with LA remodelling, specifically larger LAVi and reduced LAEF. These alterations likely reflect elevated LV filling pressures and diastolic dysfunction, which are a dominant component of the hypertensive hemodynamic response. Our findings are consistent with previous works using echocardiography. Eshoo *et al.*^27^ report large LAV in 112 patients with mild hypertension compared to 198 healthy volunteers; whilst the authors adjust for body size and sex they do not account for other comorbidities and thus cannot distinguish this remodelling pattern as distinct to hypertension.

Consistent with our biologic understanding of the hemodynamic impact of hypertension, we observed significant associations between hypertension and lower arterial compliance.^28,29^ Notably, we found that women had a greater reduction in AoD than men, suggesting that the adverse vascular effects of hypertension may be greater in women. Our study is the first to highlight this sex-differential response to hypertension.

We observed significantly lower native T1 in participants with hypertension compared to those without. This finding is consistent with a previous UK Biobank study.^30^ Existing studies^5,31^ report higher native T1 in hypertensive patients with higher degrees of LV hypertrophy (vs. hypertensives with less hypertrophy). It is not possible to directly compare these results with our analysis of hypertensive vs. non-hypertensive individuals. Furthermore, there are important technical differences in the acquisition of native T1 maps and the analysis of these images in our study compared to existing literature, which again limits direct comparisons. Possible explanations for the lower native T1 in hypertensive participants include technical factors such as reduced blood pool partial voluming with greater degrees of LV hypertrophy associated with hypertension (especially among women), differences in image segmentation methods, or discrepancies in heart rate.^32^ Biological reasons, such as an increase in the intracellular volume, might also play a role. Although typically used as a marker of diffuse interstitial fibrosis, native T1 reflects both myocyte and interstitial compartments. In pathologies such as hypertensive heart disease where both compartments are affected, native T1 should be interpreted in the appropriate context, considering disease severity, treatment effects, and adaptive remodelling patterns. Further work is required to better understand hypertension-related variations of native T1.

Our results show greater concentric LV hypertrophy, and progressively worsening diastolic dysfunction by LA metrics with increasing durations of hypertension exposure. Whilst diastolic dysfunction and impaired LV hypertrophy are well-described in the literature, this is the first study to show a dose-response relationship. In patients with a duration of exposure to hypertension of between 6 and 15 years, there was a slight improvement in LAEF. This may signify a progression of diastolic dysfunction with pseudo-normalization of the LAEF in the interim grades.^33,34^ In an echocardiography study, Inoue *et al.*^35^ found that LAEF compensated in early and middle stages of hypertension but reduced in late-stage disease. Furthermore, we found that association of hypertension with greater LVEF was only observed in patients with 11 or more years of exposure to hypertension, suggesting that this relationship is indicative of more advanced hypertension-related adaptation.

We observed remarkable attenuation of hypertension associations with adverse LV remodelling and aortic stiffness in treated hypertensives with good BP control. Chung *et al*.^36^ showed an association between poorly controlled hypertension and increased arterial stiffness, using brachial-ankle pulse wave velocity. Lønnebakken *et al.*^37^ showed that persistent LV hypertrophy was associated with suboptimal BP control. We significantly extend these observations by demonstrating attenuation of hypertension-related remodelling with optimal BP control across a range of phenotypic measures. Our observations strongly support efforts to achieve BP control in patients with hypertension.

Associations of hypertension with lower LAEF were of larger magnitude in participants with well-controlled hypertension than in those with poor control. This observation has not been previously reported in the literature and may reflect bias by indication or pharmacological mechanism of anti-hypertensives. Further studies are needed to clarify the complex relationship between anti-hypertensive medications and diastolic function.

### Limitations

The limited ethnic diversity in the UK Biobank restricts statistical power for examining associations in participants from ethnicities other than White. The analysis is limited to individuals with valid CMR metrics. Factors related to acquisition of high quality CMR images include the participants’ ability to lie flat, follow breath-hold instructions, and absence of arrhythmias (e.g. atrial fibrillation, or frequent ectopy). Given that these factors are related to poorer cardiovascular health, it is possible that we inadvertently excluded individuals with more severe adverse cardiovascular phenotypes. As this is most likely to affect participants with hypertension, the impact of this exclusion is to dampen reported associations. We present associations of hypertension in participants with different durations of exposure. Whilst this provides a representation of natural history of hypertension-related remodelling, our analysis does not include longitudinal data. Covariates, such as diabetes and high cholesterol, were treated as binary variables based on presence or absence of the diagnosis in all available linked records (primary or secondary care, self-report of the diagnosis, or self-report of relevant medications). The binary classification of disease status limits granularity of risk level, and we do not consider duration of exposure to each condition. Given that individuals with hypertension have greater propensity to cardiometabolic morbidities, incomplete capture of risk in this way may result in residual confounding with tendency towards augmentation or disruption of relationships associated with hypertension. We cannot exclude residual confounding or reverse causation.

## Conclusions

Our findings present a comprehensive assessment of cardiovascular alterations in response to hypertension providing novel insights into physiologic adaptions and variations in response to hypertension across populations.

## Supplementary data

Supplementary data are available at *European Heart Journal - Cardiovascular Imaging* online.

## Supplementary Material

jead123_Supplementary_Data

## Data Availability

This research was conducted using the UK Biobank resource under access application 2964. UK Biobank will make the data available to all bona fide researchers for all types of health-related research that is in the public interest, without preferential or exclusive access for any persons. All researchers will be subject to the same application process and approval criteria as specified by UK Biobank. For more details on the access procedure, see the UK Biobank website: http://www.ukbiobank.ac.uk/register-apply.
