# Supplementary material for: Characterizing the hypertensive cardiovascular phenotype in the UK Biobank
Source: Eur Heart J Cardiovasc Imaging. 2023 Jun 13;24(10):1352–60. doi: 10.1093/ehjci/jead123 (PMC10531143; doi:10.1093/ehjci/jead123)
Supplement: jead123_Supplementary_Data [file jead123_supplementary_data.docx]

## Supplementary Figure 1. Flow chart of participants included in the analysis


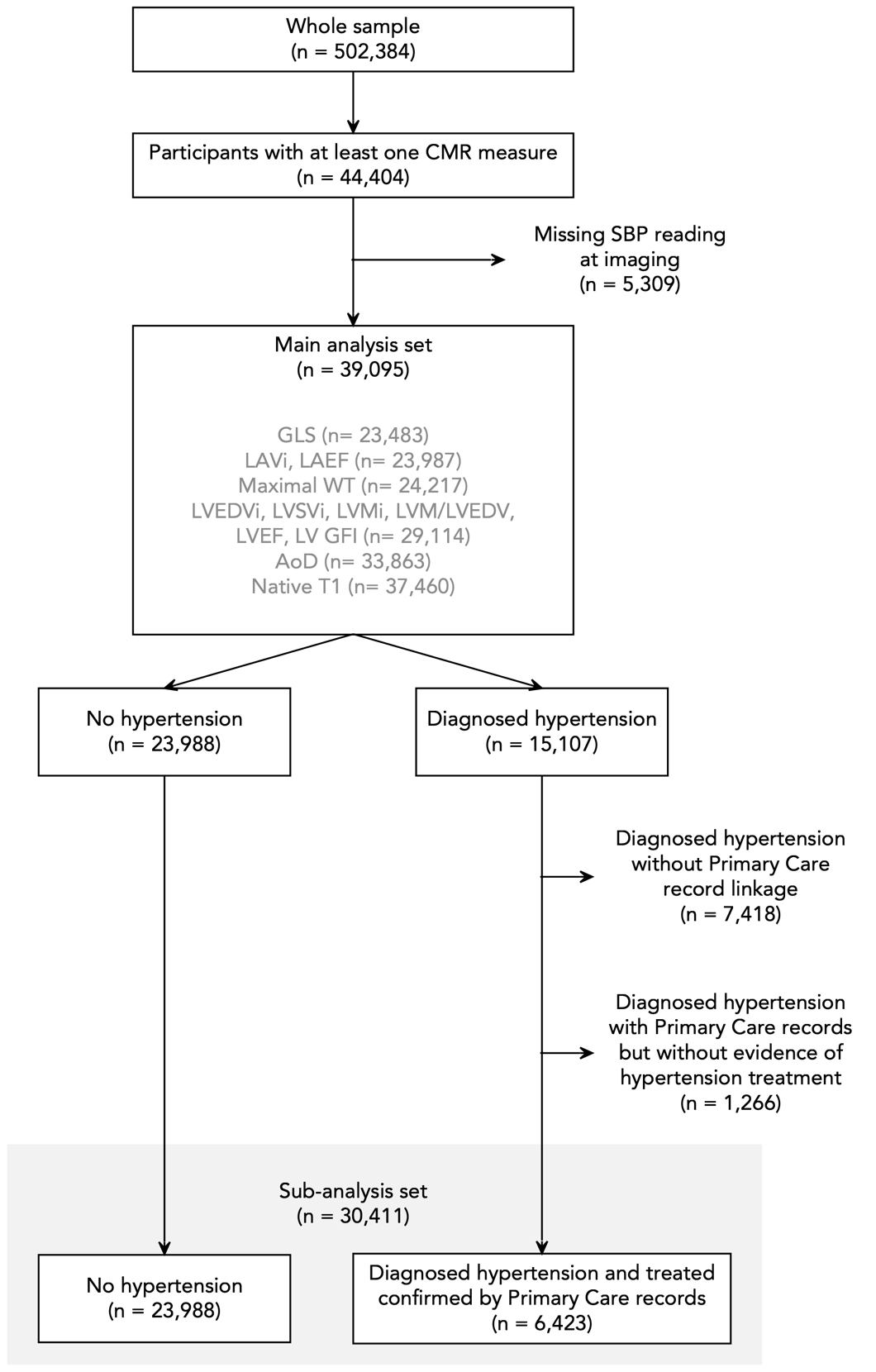


**Supplementary Figure 1 footnote.** AoD = aortic distensibility of the descending aorta, CMR= cardiovascular magnetic resonance, HTN= hypertension, GLS = Global Longitudinal Strain, LAVi = maximum left atrial volume indexed to body surface area, LAEF= left atrial ejection fraction, LVEDVi = left ventricular end-diastolic volume indexed to body surface area, LVSVi = left ventricular stroke volume indexed to body surface area, LVMi = left ventricular mass indexed to body surface area, LVM/LVEDV = left ventricular mass to volume ratio, WT= wall thickness, LVEF = left ventricular ejection fraction, LV GFI = left ventricular global function index, SBP= systolic blood pressure.

## Supplementary Table 1. Ascertainment of clinical diagnosis labels for hypertension, diabetes, and high cholesterol using HES, GP, and UK Biobank fields

| **Source** | **UKB Field ID or ICD code** | **Description** |
| --- | --- | --- |
| **Diabetes** |  |  |
| Self-report (20002) | 1220 | Diabetes |
|  | 1222 | Type 1 diabetes |
|  | 1223 | Type 2 diabetes |
| Medications (6153, 6177) | 3 | Insulin |
| Medications (20003) | 1140883066 | Insulin product |
| ICD10 | E10 | Type 1 diabetes mellitus |
|  | E11 | Type 2 diabetes mellitus |
|  | E13 | Other specified diabetes mellitus |
|  | E14 | Unspecified diabetes mellitus |
|  | G590 | Diabetic mononeuropathy |
|  | G632 | Diabetic polyneuropathy |
|  | H280 | Diabetic cataract |
|  | H360 | Diabetic retinopathy |
|  | M142 | Diabetic arthropathy |
|  | N083 | Glomerular disorders in diabetes mellitus |
|  | O240 | Diabetes mellitus in pregnancy: Pre-existing type 1 diabetes mellitus |
|  | O241 | Diabetes mellitus in pregnancy: Pre-existing type 2 diabetes mellitus |
|  | O243 | Diabetes mellitus in pregnancy: Pre-existing diabetes mellitus, unspecified |
|  | O244 | Diabetes mellitus arising in pregnancy |
|  | O249 | Diabetes mellitus in pregnancy, unspecified |
|  | Y423 | Insulin and oral hypoglycaemic [antidiabetic] drugs |
| First occurrences | 130706 | Date E10 first reported (insulin-dependent diabetes mellitus) |
|  | 130708 | Date E11 first reported (non-insulin-dependent diabetes mellitus) |
|  | 130712 | Date E13 first reported (other specified diabetes mellitus) |
|  | 130714 | Date E14 first reported (unspecified diabetes mellitus) |
| Diagnosed by doctor | 2443 | Diabetes diagnosed by doctor |
|  | 2976 | Age diabetes diagnosed by doctor |
| **High cholesterol** |  |  |
| Self-report (20002) | 1473 | High cholesterol |
| Medications (6153, 6177) | 1 | Cholesterol lowering medication |
| ICD10 | E780 | Pure hypercholesterolaemia |
|  | E782 | Mixed hyperlipidaemia |
|  | E783 | Hyperchylomicronaemia |
|  | E784 | Other hyperlipidaemia |
|  | E785 | Hyperlipidaemia, unspecified |
| First occurrences | 130814 | Date E78 first reported (disorders of lipoprotein metabolism and other lipidaemias) |
| **Hypertension** |  |  |
| Self-report (20002) | 1065 | Hypertension |
|  | 1072 | Essential hypertension |
| Medications (6153, 6177) | 2 | Blood pressure medication |
| ICD10 | I10 | Essential (primary) hypertension |
| First occurrences | 131286 | Date I10 first reported (essential (primary) hypertension) |
| Diagnosed by doctor | 6150: 4 | High blood pressure |
|  | 2966 | Age high blood pressure diagnosed |
| GP Clinical records  (42040) | ctv | .14A2, .6627, .6628, .662d, .662F, .662O, .662P, .G3.., .G31., .G35., .G36., 14A2., 662d., 662F., 662O., 662P., 662P0, G200., G201., G203., G24.., G240., G24z., G24zz, G26.., G28.., Xa3fQ, Xa8HD, XaIyE, XaXOi, XaZbz, XaZWn, XE0Ub, XE0Uc, XE0Ud, XE0W8, XM1YA, XSDSb |
|  | emis | EMISHGT69, EMISNQST25, HNGZ016 |

**Supplementary Table 1 footnote**: ICD10 codes are drawn from fields 41270, 41280, 41234 and 41259; Where a 3-digit code is given, this includes all 4-digit sub-codes, for example, E10 includes E100, E101 and E102. GP: general practice; HES= Hospital Episode Statistics, ICD= international classification of disease.

## Supplementary Table 2. List of antihypertensive medications included in defining “treated hypertension”

| **Code type** | **Code** |
| --- | --- |
| BNF codes  (GP prescription records, 42039) | 02.02.01.00.00, 02.02.03.00.00, 02.02.04.00.00, 02.02.08.00.00, 02.03.02.02.00, 02.04.00.00.00, 02.05.04.00.00, 02.05.05.01.00, 02.05.05.02.00, 02.06.02.00.00, 0202010B0BEAAAC, 0202010D0AAAFAF, 0202010F0BBAAAA, 0202010L0AAABAB, 0202010L0AAACAC, 0202010P0AAAAAA, 0202010P0AAADAD, 0202030S0AAATAT, 0202030S0AAAUAU, 0202030S0AAAVAV, 0202040A0BBAAAA, 0202040C0BDAAAA, 0202040C0BDABAC, 0202040C0BIAAAB, 0202040H0BBAAAA, 0202040V0BBAAAA, 020400060AAAAAA, 020400060AAABAB, 020400060BBAAAA, 020400080AAABAB, 020400080AAACAC, 020400080AAAEAE, 020400080AAAFAF, 0204000C0AAABAB, 0204000E0AAAAAA, 0204000E0AAABAB, 0204000E0AAACAC, 0204000E0AAAGAG, 0204000E0BBACAC, 0204000E0BBAEAG, 0204000E0BBAFAB, 0204000H0AAAAAA, 0204000H0AAABAB, 0204000H0BBAAAA, 0204000H0BBABAB, 0204000H0BCAAAA, 0204000H0BCABAB, 0204000I0AAACAC, 0204000I0AAADAD, 0204000I0AAAEAE, 0204000K0AAABAB, 0204000K0AAACAC, 0204000K0AAADAD, 0204000N0AAABAB, 0204000N0AAACAC, 0204000R0AAAAAA, 0204000R0AAABAB, 0204000R0AAAHAH, 0204000R0AAAJAJ, 0204000R0AAAKAK, 0204000R0AAALAL, 0204000R0AAAYAY, 0204000R0BGAAAH, 0204000R0BGABAJ, 0204000R0BGACAK, 0204000R0BGAFAA, 0204000R0BGAGAB, 0204000R0BNABAA, 0204000U0BBAAAA, 0204000U0BCAAAA, 0205040D0AAAAAA, 0205040D0AAABAB, 0205040D0AAACAC, 0205040D0BBABAB, 0205040D0BBACAC, 0205040M0AAACAC, 0205040S0AAABAB, 0205040S0AAACAC, 0205040S0AAADAD, 0205040V0BBABAB, 0205051F0AAADAD, 0205051F0AAAEAE, 0205051F0AAAFAF, 0205051F0BCABAE, 0205051H0BBAAAA, 0205051I0AAAAAA, 0205051I0AAABAB, 0205051I0AAACAC, 0205051I0AAADAD, 0205051I0BBABAB, 0205051I0BBACAC, 0205051I0BBADAD, 0205051J0AAAAAA, 0205051J0AAABAB, 0205051J0BBAAAA, 0205051J0BBABAB, 0205051K0BCAAAA, 0205051K0BCABAB, 0205051L0AAAAAA, 0205051L0AAABAB, 0205051L0AAACAC, 0205051L0AAADAD, 0205051L0BBABAB, 0205051L0BBACAC, 0205051L0BBADAD, 0205051L0BCABAB, 0205051L0BCACAC, 0205051M0AAAAAA, 0205051M0AAABAB, 0205051Q0AAAAAA, 0205051Q0AAABAB, 0205051Q0AAACAC, 0205051Q0AAADAD, 0205051R0AAAAAA, 0205051R0AAABAB, 0205051R0AAACAC, 0205051U0AAAAAA, 0205051U0AAABAB, 0205051U0AAACAC, 0205051U0BBAAAA, 0205052C0AAAAAA, 0205052C0AAABAB, 0205052C0AAACAC, 0205052C0AAADAD, 0205052C0BBABAB, 0205052I0AAAAAA, 0205052I0AAABAB, 0205052I0AAACAC, 0205052I0BBAAAA, 0205052I0BBABAB, 0205052N0AAAAAA, 0205052N0AAABAB, 0205052N0BBABAB, 0205052P0BBAAAA, 0205052V0AAAAAA, 0205052V0AAABAB, 0205052V0AAACAC, 0205052V0BBAAAA, 0205052V0BBABAB, 0205052V0BBACAC, 0206020A0AAAAAA, 0206020A0AAABAB, 0206020A0BBAAAA, 0206020A0BBABAB, 0206020C0AAAAAA, 0206020C0AAACAC, 0206020C0AAAEAE, 0206020C0AAAJAJ, 0206020C0AAASAS, 0206020C0AAATAT, 0206020C0AAAUAU, 0206020C0AAAVAV, 0206020C0AAAXAX, 0206020C0AAAYAY, 0206020C0BBAAAA, 0206020C0BBABAC, 0206020C0BBACAS, 0206020C0BBADAE, 0206020C0BBAEAX, 0206020C0BFAFAE, 0206020C0BFAGAU, 0206020C0BFAHAV, 0206020C0BFAIAW, 0206020C0BHAAAJ, 0206020C0BHABAT, 0206020C0BHADAU, 0206020C0BHAEAV, 0206020C0BHAFAW, 0206020C0BIAAAU, 0206020C0BIABAV, 0206020C0BIACAW, 0206020C0BJAAAT, 0206020C0BJABAU, 0206020F0AAABAB, 0206020F0AAACAC, 0206020F0AAADAD, 0206020K0AAAAAA, 0206020K0AAABAB, 0206020K0BBAAAA, 0206020K0BBABAB, 0206020L0AAAAAA, 0206020L0BBAAAA, 0206020Q0AAACAC, 0206020R0AAAAAA, 0206020R0AAABAB, 0206020R0AAAEAE, 0206020R0AAAHAH, 0206020R0AAANAN, 0206020R0AAAPAP, 0206020R0AAARAR, 0206020R0AAAXAX, 0206020R0BBAAAA, 0206020R0BBABAB, 0206020R0BBAFAR, 0206020R0BBAGAE, 0206020R0BBAHAN, 0206020R0BBAIAP, 0206020R0BGAAAH, 0206020R0BGABAM, 0206020R0BRAAAR, 0206020R0BRABAE, 0206020T0AAACAC, 0206020T0AAADAD, 0206020T0AAAFAF, 0206020T0AAAGAG, 0206020T0AAAHAH, 0206020T0AAAIAI, 0206020T0AAAJAJ, 0206020T0AAAKAK, 0206020T0AAAUAU, 0206020T0BDAAAC, 0206020T0BDAEAH, 0206020T0BDAGAU, 04.07.04.02.00, 04.09.03.00.00, 06.02.02.00.00, 07.04.01.01.00, 0704010M0AAAAAA, 0704010M0BBAAAA, 0704010T0AAAAAA, 0704010T0AAABAB, 0704010T0AAACAC, 0704010T0AAADAD, 0704010T0AAAEAE, 0704010T0BBACAC, 0704010T0BBAEAE, 1.1922853254726199E-288, 1.5641417457509001E-291, 1.82173938119823E-295, 1.8304310759580301E-295, 1.99219498092525E-296, 1.9949111355376799E-296, 10704, 2.2625790598526402E-308, 2.3368617505322202E-305, 2.6462641299971701E-304, 2.6540533138508801E-302, 20201, 20203, 20208, 204, 20504, 2050501, 2050502, 20602, 3.14246421774018E-301, 3.6091549775929602E-308, 3.7271159993844801E-296, 3.7967653921777399E-298, 3.80100938375967E-298, 4.0433755138831602E-303, 4.4258369088462901E-302, 4.5287059189769701E-304, 4.7988418242186503E-307, 4.8081455351338203E-296, 5.22643526561921E-308, 50.00.00.00.00, 6.24984284441478E-308, 7.4233992089281596E-292, 8.7794961788472397E-293, 8.7906215481397701E-293, 9.4465019797994697E-299, None |
| Read2 or ctv codes   (GP clinical or prescription records, 42039 or 42040) | .8B26, .8B6Q, .8B6T, .8BL0, 7Q01., 7Q01z, 8B26., 8B6E., 8B6F2, 8B6F3, 8B6G., 8B6H., 8B6Q., 8B6R., 8B6T., 8BG4., 8BGA., 8BL0., 8BMa1, b21.., b211., b211.00, b212., b212.00, b213., b213.00, b214., b214.00, b215., b216., b217., b218., b219., b21b., b22.., b221., b221.00, b22y., b22z., b22z.00, b231., b231.00, b232., b23z., b25.., b251., b251.00, b25z., b26.., b261., b262., b263., b264., b26y., b26z., b27.., b27z., b28.., b285., b285.00, b28z., b28z.00, b2a.., b2az., b2b.., b2b..00, b2b1., b2b2., b2b3., b2bz., b2bz.00, b2c.., b2c1., b2cz., b414., b43.., b431., b431.00, b432., b432.00, b433., b433.00, b434., b434.00, b435., b436., b43a., b43A., b43b., b43c., b43d., b43e., b43f., b43g., b43h., b43j., b43k., b43l., b43m., b43m.00, b43n., b43z., b43z.00, b511., b512., b513., b514., b514.00, b515., b518., b51a., b51a.00, b51A.00, b51b., b51b.00, b51c., b51C.00, b51E., b51f., b51F., b51G., b51J., b51j.00, b51J.00, b51K., b51k.00, b51p.00, b51r., b51r.00, b51s., b51v., b51w., b913., b913.00, b915., b916., b919., b919.00, b91a., b91a.00, b91c., b91e., b91h., b91h.00, bA…, bA1.., bA11., bA12., bA1y., bA1z., bA1z.00, bb3.., bb31., bb31.00, bb32., bb32.00, bb33., bb33.00, bb34., bb35., bb36., bb37., bb38., bb39., bb3a., bb3A., bb3A.00, bb3b., bb3B., bb3B.00, bb3C., bb3C.00, bb3d., bb3D., bb3e., bb3f., bb3F., bb3g., bb3g.00, bb3h., bb3i., bb3j., bb3j.00, bb3k., bb3k.00, bb3M., bb3M.00, bb3p., bb3q., bb3r., bb3v., bb3v.00, bb3w., bb3w.00, bb3y., bb3y.00, bb3z., bb3z.00, bd…, bd1.., bd11., bd11.00, bd12., bd12.00, bd13., bd13.00, bd14., bd14.00, bd15., bd16., bd17., bd18., bd19., bd1a., bd1A., bd1b., bd1B., bd1c., bd1C., bd1D., bd1E., bd1F., bd1G., bd1h., bd1i., bd1I., bd1j., bd1J., bd1k., bd1K., bd1l., bd1L., bd1l.00, bd1L.00, bd1m., bd1m.00, bd1n., bd1n.00, bd1o., bd1o.00, bd1p., bd1P., bd1p.00, bd1Q., bd1r., bd1R., bd1r.00, bd1S., bd1t., bd1T., bd1u., bd1U., bd1v., bd1V., bd1w., bd1W., bd1W.00, bd1x., bd1X., bd1x.00, bd1y., bd1Y., bd1y.00, bd2.., bd21., bd22., bd23., bd2w., bd2w.00, bd2x., bd2x.00, bd2y., bd3.., bd31., bd31.00, bd32., bd34., bd34.00, bd35., bd35.00, bd36., bd36.00, bd37., bd38., bd38.00, bd39., bd3c., bd3c.00, bd3d., bd3e., bd3j., bd3j.00, bd3x., bd3x.00, bd5.., bd51., bd51.00, bd52., bd52.00, bd53., bd5t., bd5u., bd5v., bd5w., bd5x., bd5y., bd6.., bd6w., bd6w.00, bd6x., bd6x.00, bd6z., bd6z.00, bd8.., bd81., bd82., bd82.00, bd83., bd84., bd84.00, bd8u., bd8u.00, bd9.., bdd.., bdd1., bdd2., bddz., bde1., bde2., bde3., bde4., bde5., bde6., bde7., bde7.00, bde9., bdea., bdeA., bdeA.00, bdeb., bdeB., bdec., bdeC., bdeD., bdeE., bdeF., bdej., bdej.00, bdek., bdeK., bdeK.00, bdeL., bdeL.00, bdem., bdeM., bdem.00, bden.00, bdeo., bdep., bdeP., bdeP.00, bdeq., bder., bdes., bdes.00, bdet., bdet.00, bdeu.00, bdev., bdev.00, bdew., bdex., bdey., bdey.00, bdez., bdez.00, bdf.., bdf1., bdf1.00, bdf2., bdf2.00, bdf3., bdf3.00, bdf4., bdf4.00, bdf5., bdf5.00, bdf6., bdf8., bdf9., bdf9.00, bdfA., bdfA.00, bdfB., bdfC., bdfC.00, bdfD., bdfE., bdfE.00, bdfw., bdfw.00, bdfx., bdfx.00, bdfy., bdfy.00, bdfz., bdfz.00, bdh.., bdh1., bdh2., bdi.., bdi1., bdj.., bdj1., bdj1.00, bdj2., bdj3., bdj3.00, bdj4., bdj4.00, bdj5., bdj5.00, bdl.., bdl3., bdl3.00, bdl4., bdl4.00, bdl5., bdl5.00, bdl6., bdl6.00, bdm.., bdm..00, bdm1., bdm1.00, bdmy., bdmy.00, bdmz., bdmz.00, bdn.., bdn6., be…, bf…, bf3c., bh1.., bh11., bh12., bh13., bh13.00, bh14., bh14.00, bh1y., bh1y.00, bh1z., bh2.., bh21., bh22., bh2y., bh2y.00, bh2z., bh4.., bh41., bh41.00, bh42., bh42.00, bh43., bh44., bh45., bh46., bh47., bh48., bh49., bh4v., bh4v.00, bh4w., bh4w.00, bh4x., bh4x.00, bh4y., bh4y.00, bh4z., bh4z.00, bh5.., bh51., bh51.00, bh52., bh52.00, bh53., bh53.00, bh54., bh54.00, bh55., bh55.00, bh56., bh56.00, bh57., bh5x., bh5y., bh5z., bh5z.00, bh6.., bh61., bh61.00, bh62., bh62.00, bh63., bh63.00, bh64., bh64.00, bh65., bh65.00, bh66., bh66.00, bh67., bh67.00, bh68., bh68.00, bh6y., bh6y.00, bh6z., bh6z.00, bi…, bi1.., bi11., bi12., bi13., bi14., bi15., bi16., bi16.00, bi17., bi18., bi18.00, bi19., bi19.00, bi1a., bi1A., bi1a.00, bi1B., bi1C., bi1G.00, bi1I., bi1K., bi1p., bi1q., bi1r., bi1v., bi1v.00, bi1w., bi1x., bi1x.00, bi1y., bi1z., bi1z.00, bi2.., bi21., bi21.00, bi22., bi22.00, bi23., bi23.00, bi24., bi24.00, bi25., bi25.00, bi26., bi27., bi27.00, bi28., bi28.00, bi29., bi2a., bi2A., bi2a.00, bi2b., bi2B., bi2b.00, bi2C., bi2D., bi2E., bi2F., bi2G., bi2H., bi2t., bi2t.00, bi2u., bi2u.00, bi2v., bi2v.00, bi2w., bi2w.00, bi2x., bi2x.00, bi2y., bi2y.00, bi2z., bi2z.00, bi3.., bi31., bi31.00, bi32., bi32.00, bi33., bi33.00, bi34., bi34.00, bi35., bi36., bi37., bi38., bi39., bi3a., bi3b., bi3c., bi3d., bi3e., bi3f., bi3g., bi3h., bi3i., bi3j., bi3k., bi3l., bi3p., bi3p.00, bi3q., bi3r., bi3t., bi3t.00, bi3y., bi4.., bi41., bi41.00, bi42., bi42.00, bi43., bi43.00, bi47., bi48., bi48.00, bi4A., bi4A.00, bi4F., bi5.., bi51., bi51.00, bi52., bi52.00, bi53., bi53.00, bi54., bi54.00, bi55., bi55.00, bi56., bi56.00, bi57., bi57.00, bi58., bi58.00, bi6.., bi6..00, bi61., bi61.00, bi62., bi62.00, bi63., bi63.00, bi67., bi67.00, bi69., bi69.00, bi6B., bi6B.00, bi6C., bi6C.00, bi6D., bi6D.00, bi6E., bi6E.00, bi6F., bi6F.00, bi6G., bi7.., bi71., bi71.00, bi72., bi72.00, bi73., bi73.00, bi74., bi74.00, bi8.., bi81., bi81.00, bi82., bi83., bi84., bi85., bi86., bi87., bi88., bi89., bi8a., bi8a.00, bi9.., bi91., bi91.00, bi92., bi92.00, bi93., bi93.00, bi94., bi95., bi95.00, bi96., bi96.00, bi97., bi98., bi99., bi9A., bi9z., bi9z.00, biA.., biA1., biA2., biA3., biA4., biB.., biB1., biB2., biB3., biBx., biBx.00, biBy., biBz., biBz.00, biC.., biC1., biC2., biC2.00, biC3., biC4., biC4.00, biC5., biC6., biC6.00, biC7., biC7.00, biC8., biC8.00, bk…, bk2.., bk3.., bk31., bk31.00, bk32., bk32.00, bk33., bk33.00, bk34., bk34.00, bk35., bk35.00, bk36., bk36.00, bk37., bk37.00, bk38., bk38.00, bk39., bk39.00, bk3A., bk3B., bk3C., bk3C.00, bk3D., bk3E., bk3y., bk3y.00, bk3z., bk3z.00, bk4.., bk41., bk41.00, bk42., bk42.00, bk43., bk43.00, bk44., bk44.00, bk45., bk45.00, bk46., bk46.00, bk47., bk47.00, bk48., bk48.00, bk49., bk49.00, bk4A., bk4B., bk4C., bk4s., bk4s.00, bk4t., bk4t.00, bk4u., bk4v., bk4v.00, bk4w., bk4w.00, bk4x., bk4x.00, bk4y., bk4y.00, bk4z., bk4z.00, bk5.., bk51., bk51.00, bk52., bk52.00, bk53., bk53.00, bk54., bk54.00, bk55., bk55.00, bk56., bk56.00, bk57., bk57.00, bk58., bk58.00, bk59., bk59.00, bk5x., bk5y., bk5y.00, bk5z., bk5z.00, bk6.., bk61., bk61.00, bk7.., bk71., bk71.00, bk72., bk72.00, bk73., bk73.00, bk74., bk74.00, bk75., bk75.00, bk76., bk76.00, bk77., bk77.00, bk78., bk78.00, bk79., bk79.00, bk7z., bk7z.00, bk8.., bk81., bk81.00, bk82., bk82.00, bk86., bk86.00, bk87., bk88., bk8w., bk8x., bk8x.00, bk8y., bk8y.00, bk8z., bk8z.00, bk9.., bk91., bk91.00, bk92., bk93., bk9x., bk9x.00, bk9y., bk9y.00, bk9z., bk9z.00, bkB.., bkB1., bkB1.00, bkB2., bkB2.00, bkB3., bkB3.00, bkB4., bkB4.00, bkB5., bkB5.00, bkB6., bkC.., bkC1., bkC2., bkC2.00, bkC3., bkCx., bkCx.00, bkCy., bkCy.00, bkCz., bkCz.00, bkD.., bkD1., bkD1.00, bkD2., bkD2.00, bkD3., bkD3.00, bkH.., bkHx., bkHy., bkHy.00, bkHz., bkHz.00, bkI.., bl5.., bl51., bl51.00, bl53., bl55., bl55.00, bl56., bl57., bl58., bl58.00, bl59., bl59.00, bl5a., bl5A., bl5a.00, bl5A.00, bl5b., bl5b.00, bl5c., bl5c.00, bl5d., bl5d.00, bl5e., bl5E., bl5e.00, bl5E.00, bl5f., bl5F., bl5f.00, bl5F.00, bl5g., bl5G., bl5g.00, bl5G.00, bl5h., bl5H., bl5h.00, bl5H.00, bl5i., bl5I., bl5I.00, bl5j., bl5J., bl5J.00, bl5k., bl5K., bl5K.00, bl5L., bl5L.00, bl5m., bl5M., bl5M.00, bl5n., bl5N., bl5N.00, bl5O., bl5O.00, bl5P., bl5P.00, bl5Q., bl5Q.00, bl5R., bl5R.00, bl5s., bl5S., bl5s.00, bl5S.00, bl5U., bl5U.00, bl5x., bl5x.00, bl5y., bl5y.00, bl5z., bl5Z., bl5z.00, bl5Z.00, bl7.., bl71., bl71.00, bl72., bl72.00, bl73., bl73.00, bl74., bl7w., bl7w.00, bl7x., bl7x.00, bl7y., bl7y.00, bl7z., bl7z.00, bl8.., bl8..00, bl81., bl81.00, bl82., bl82.00, bl83., bl83.00, bl84., bl84.00, bl85., bl85.00, bl86., bl86.00, bl8A., bl8A.00, bl8b., bl8B., bl8B.00, bl8e., bl8e.00, bl8f., bl8F., bl8F.00, bl8g., bl8G., bl8h., bl8i., bl8i.00, bl8j., bl8j.00, bl8k., bl8k.00, bl8m., bl8M., bl8M.00, bl8P., bl8Q., bl8R., bl8u., bl8u.00, bl8v., bl8V., bl8v.00, bl8V.00, bl8w., bl8W., bl8w.00, bl8W.00, bl8x., bl8X., bl8x.00, bl8X.00, bl8y., bl8Y., bl8y.00, bl8Y.00, bl8z., bl8z.00, bla.., bla1., bla1.00, bla2., blb.., blb1., blb1.00, blb2., blb2.00, blb3., blb3.00, blb4., blb4.00, blb7., blb8., blc.., blc1., blc1.00, blc2., blc2.00, blc5., blc5.00, blci., blci.00, blcj., blcj.00, ble.., ble1., ble1.00, ble2., ble2.00, ble3., ble3.00, ble4., ble4.00, blh.., blh1., blh1.00, blh2., blh2.00, blh3., blh3.00, blh4., blj.., blj1., blj1.00, blj2., blj3., blja., blja.00, bljJ., bljJ.00, bljP., bljP.00, bljY., bljY.00, bljZ., bll.., bll3., bll4., bllc., bllc.00, blld., blld.00, gc41., gc5.., gc51., gc51.00, gc52., gc53., gc54., gc55., gc5z., gc5z.00, k85.., x0004, x000z, x0010, x001j, x0044, x007H, x007i, x007v, x007w, x008F, x008y, x009B, x00A3, x00B5, x00B6, x00Bb, x00BL, x00Bu, x00Du, x00Te, x00Tl, x00Tm, x01C1, x01C2, x01C4, x01C6, x01C7, x01CA, x01CB, x01CD, x01CE, x01CF, x01CJ, x01CK, x01CN, x01CO, x01CP, x01CR, x01CS, x01CT, x01CU, x01CV, x01CW, x01Qe, x01Qf, x01Qg, x01QU, x01QV, x01QW, x01QX, x02ao, x02aQ, x02at, x02bC, x02bD, x02eC, x02eO, x02eP, x02fa, x02fB, x02fC, x02fG, x02fH, x02fI, x02fl, x02fm, x02fW, x02fX, x02fY, x02fz, x02fZ, x02g6, x02g7, x02im, x02jh, x02ji, x02jj, x02jn, x02jv, x02ke, x02kf, x02kk, x02kW, x02kX, x02lk, x02o9, x02oA, x02oB, x02oV, x02oW, x02Qa, x02Qc, x02Qd, x02Qe, x02qg, x02Qg, x02qh, x02Qh, x02QH, x02Qi, x02QI, x02Qj, x02QJ, x02Qk, x02QK, x02QL, x02QM, x02QO, x02qQ, x02QS, x02QW, x02QY, x02QZ, x02rM, x02rO, x02rP, x02rQ, x02rR, x02rU, x02Sc, x02Sd, x02sK, x02sL, x02Sn, x02So, x02sP, x02sQ, x02T4, x02T5, x02t6, x02tB, x02Tb, x02tC, x02Tc, x02TQ, x02Ty, x02u7, x02UA, x02UD, x02Ug, x02um, x02uP, x02Uy, x02Vc, x02Vm, x02VN, x02VO, x02VQ, x02VR, x02VT, x02VV, x02Wf, x02Wj, x02Wk, x02Wn, x02Wo, x02Wp, x02Wq, x02X3, x02Xf, x02Xg, x02Y2, x02Y4, x02YD, x02YE, x02Yx, x02Za, x03ce, x03df, x03e5, x03h1, x03iy, x03j2, x03jn, x03jo, x03ls, x03lt, x03n3, x03pa, x03qe, x03qf, x03qX, x03vs, x03vt, x03wm, x03xa, x03yR, x03zU, x0411, x0412, x046e, x046f, x047a, x047f, x047g, x047h, x047r, x047s, x047Y, x047Z, x048y, x048z, x049y, x04bN, x04bO, x04bR, x04c7, x04qm, x04qn, x04t2, x04t3, x04t4, x04t8, x04t9, x04tA, x04tB, x04vd, x04vg, x04xj, x04y0, x04yc, x04yd, x051w, x051x, x056B, x056P, x05DC, x05df, x05dg, x05Dq, x05F5, x05hA, x05j2, x05mo, x05rz, x05u2, x05wO, x060W, XaBLq, XaJ5h, XaM5f, XaM5p, [emis] EMISQPR5 |
| Self-reported medications  (UKB Field 20003) | 1140860334, 1140860336, 1140860338, 1140860340, 1140860342, 1140860348, 1140860352, 1140860356, 1140860358, 1140860380, 1140860382, 1140860386, 1140860390, 1140860394, 1140860396, 1140860398, 1140860402, 1140860404, 1140860406, 1140860410, 1140860418, 1140860422, 1140860426, 1140860434, 1140860492, 1140860498, 1140860562, 1140860564, 1140860580, 1140860590, 1140860610, 1140860654, 1140860658, 1140860690, 1140860696, 1140860706, 1140860714, 1140860728, 1140860736, 1140860738, 1140860750, 1140860752, 1140860758, 1140860764, 1140860776, 1140860784, 1140860790, 1140860802, 1140860806, 1140860878, 1140860882, 1140860892, 1140860904, 1140860912, 1140860918, 1140861088, 1140861090, 1140861106, 1140861110, 1140861114, 1140861120, 1140861128, 1140861130, 1140861136, 1140861138, 1140861166, 1140861176, 1140861190, 1140861194, 1140861202, 1140861276, 1140861282, 1140864950, 1140864952, 1140866072, 1140866078, 1140866090, 1140866092, 1140866094, 1140866096, 1140866102, 1140866104, 1140866122, 1140866128, 1140866132, 1140866136, 1140866138, 1140866140, 1140866144, 1140866146, 1140866156, 1140866158, 1140866162, 1140866164, 1140866168, 1140866226, 1140866232, 1140866236, 1140866244, 1140866262, 1140866306, 1140866308, 1140866312, 1140866318, 1140866324, 1140866328, 1140866330, 1140866340, 1140866352, 1140866354, 1140866360, 1140866396, 1140866400, 1140866402, 1140866404, 1140866410, 1140866416, 1140866420, 1140866440, 1140866446, 1140866450, 1140866460, 1140866466, 1140866484, 1140866546, 1140866554, 1140866692, 1140866704, 1140866712, 1140866724, 1140866726, 1140866738, 1140866756, 1140866758, 1140866764, 1140866766, 1140866778, 1140866782, 1140866784, 1140866798, 1140866800, 1140866802, 1140866804, 1140879760, 1140879762, 1140879778, 1140879782, 1140879786, 1140879794, 1140879798, 1140879802, 1140879806, 1140879810, 1140879818, 1140879824, 1140879830, 1140879834, 1140879842, 1140879866, 1140888510, 1140888552, 1140888556, 1140888560, 1140888578, 1140888646, 1140909368, 1140911698, 1140916356, 1140916362, 1140917428, 1140923572, 1140923712, 1140923718, 1140926778, 1140926780, 1141145658, 1141145660, 1141145668, 1141151016, 1141151018, 1141151382, 1141152600, 1141152998, 1141153006, 1141153026, 1141153032, 1141153328, 1141156754, 1141156808, 1141156836, 1141156846, 1141164148, 1141164154, 1141164276, 1141164280, 1141165470, 1141165476, 1141166006, 1141167822, 1141167832, 1141171152, 1141171336, 1141171344, 1141172682, 1141172686, 1141180592, 1141180598, 1141187788, 1141187790, 1141190160, 1141193282, 1141193346, 1141194794, 1141194800, 1141194804, 1141194808, 1141194810, 1141201038, 1141201040 |

## Supplementary Table 3. Summary of CMR metrics by hypertension status

|  | Whole sample | Diagnosed Hypertension | No Hypertension | Overall N for  each metric |
| --- | --- | --- | --- | --- |
| LAVi | 38.1 [31.5, 45.5] | 38.8 [31.7, 46.8] | 37.7 [31.3, 44.7] | 23,987 |
| LAEF | 61.3 (±9.1) | 60.1 (±10.3) | 62.0 (±8.1) | 23,987 |
| LVEDVi | 79.3 (±14.1) | 79.3 (±14.7) | 79.3 (±13.6) | 29,114 |
| LVSVi | 46.9 (±8.4) | 46.8 (±8.6) | 47.0 (±8.3) | 29,114 |
| LVMi | 46.0 (±8.7) | 48.0 (±9.1) | 44.8 (±8.3) | 29,114 |
| LVM/LVEDV | 0.57 [0.52, 0.64] | 0.60 [0.55, 0.67] | 0.56 [0.51, 0.61] | 29,114 |
| Maximal WT | 7.1 (±1.1) | 7.4 (±1.1) | 6.9 (±1.0) | 24,217 |
| LVEF | 59.5 (±6.1) | 59.4 (±6.6) | 59.5 (±5.7) | 29,114 |
| LV GFI | 0.48 (±0.07) | 0.47 (±0.07) | 0.48 (±0.07) | 29,114 |
| GLS | -18.5 (±2.7) | -18.3 (±2.9) | -18.5 (±2.6) | 23,483 |
| Native T1 | 931.8 (±35.7) | 928.4 (±35.3) | 934.0 (±35.8) | 37,460 |
| AoD | 2.15 [1.53, 2.98] | 1.89 [1.37, 2.57] | 2.34 [1.66, 3.21] | 34,500 |

**Supplementary Table 3 footnote**. CMR= cardiovascular magnetic resonance, LAVi = maximum left atrial volume indexed to body surface area, LAEF= left atrial ejection fraction, LVEDVi = left ventricular end-diastolic volume indexed to body surface area, LVSVi = left ventricular stroke volume indexed to body surface area, LVMi = left ventricular mass indexed to body surface area, LVM/LVEDV = left ventricular mass to volume ratio, WT= wall thickness, LVEF = left ventricular ejection fraction, LV GFI = left ventricular global function index, AoD = aortic distensibility of the descending aorta.

## Supplementary Table 4. Associations of systolic blood pressure with CMR metrics in linear regression models

| **Metric** | **Crude association in whole sample** | **Fully adjusted in whole sample** | **Fully adjusted in women (n= 20,138)** | **Fully adjusted in  men (n= 18,957)** |
| --- | --- | --- | --- | --- |
| LAVi | 0.036* [0.029, 0.043] | 0.045* [0.037, 0.052] | 0.057* [0.047, 0.066] | 0.033* [0.021, 0.045] |
|  | 5.87x10-24 | 9.80x10-31 | 8.47x10-31 | 1.00x10-7 |
| LAEF | -0.011* [-0.018, -0.004] | 0.027* [0.019, 0.034] | 0.005 [-0.005, 0.014] | 0.051* [0.039, 0.063] |
|  | 0.0025 | 5.09x10-12 | 0.3536 | 2.00x10-17 |
| LVEDVi | 0.032* [0.026, 0.038] | 0.040* [0.034, 0.047] | 0.042* [0.035, 0.049] | 0.039* [0.028, 0.049] |
|  | 3.76x10-23 | 8.04x10-38 | 8.80x10-31 | 2.60x10-13 |
| LVSVi | 0.038* [0.031, 0.044] | 0.062* [0.056, 0.069] | 0.054* [0.046, 0.062] | 0.071* [0.061, 0.082] |
|  | 9.41x10-32 | 1.03x10-77 | 6.28x10-41 | 2.31x10-39 |
| LVMi | 0.176* [0.170, 0.182] | 0.134* [0.129, 0.139] | 0.122* [0.116, 0.128] | 0.147* [0.138, 0.156] |
|  | < 1.00x10-250 | < 1.00x10-250 | < 1.00x10-250 | 2.24x10-228 |
| LVM/LVEDV | 0.181* [0.175, 0.187] | 0.116* [0.110, 0.122] | 0.109* [0.102, 0.116] | 0.123* [0.113, 0.132] |
|  | < 1.00x10-250 | 1.91x10-320 | 5.98x10-192 | 5.01x10-136 |
| Maximal WT | 0.218* [0.212, 0.225] | 0.128* [0.123, 0.134] | 0.121* [0.115, 0.128] | 0.134* [0.126, 0.143] |
|  | < 1.00x10-250 | < 1.00x10-250 | 2.96x10-286 | 2.35x10-191 |
| LVEF | 0.017* [0.011, 0.024] | 0.043* [0.036, 0.049] | 0.028* [0.020, 0.037] | 0.059* [0.049, 0.069] |
|  | 5.84x10-8 | 3.05x10-37 | 6.35x10-11 | 2.36x10-29 |
| LV GFI | -0.070* [-0.076, -0.064] | -0.018* [-0.025, -0.012] | -0.029* [-0.038, -0.021] | -0.004 [-0.013, 0.005] |
|  | 1.63x10-106 | 6.79x10-9 | 1.17x10-11 | 0.3717 |
| GLS | 0.019* [0.012, 0.026] | 0.003 [-0.004, 0.011] | 0.007 [-0.003, 0.017] | -0.001 [-0.012, 0.010] |
|  | 1.50x10-7 | 0.3939 | 0.1938 | 0.8442 |
| Native T1 | -0.080* [-0.085, -0.074] | -0.051* [-0.056, -0.045] | -0.058* [-0.065, -0.050] | -0.033* [-0.041, -0.025] |
|  | 5.19x10-184 | 5.47x10-73 | 2.93x10-51 | 6.86x10-16 |
| AoD | -0.225* [-0.231, -0.220] | -0.163* [-0.168, -0.158] | -0.170* [-0.177, -0.163] | -0.151* [-0.158, -0.144] |
|  | < 1.00x10-250 | < 1.00x10-250 | < 1.00x10-250 | < 1.00x10-250 |

**Supplementary Table 4 footnote.** Results are standardised beta coefficients and 95% CIs with corresponding p-values, representing SD change in CMR metrics associated with every 10mmHg increase in systolic BP. Fully adjusted models include adjustment for age, sex, ethnicity, Townsend deprivation index, alcohol, BMI, smoking, diabetes, high cholesterol. An * indicates p-values that were significant after adjustment for multiple testing with a false discovery rate of 5%. BP= blood pressure, CI= confidence interval, CMR= cardiovascular magnetic resonance, LAVi = maximum left atrial volume indexed to body surface area, LAEF= left atrial ejection fraction, LVEDVi = left ventricular end-diastolic volume indexed to body surface area, LVSVi = left ventricular stroke volume indexed to body surface area, LVMi = left ventricular mass indexed to body surface area, LVM/LVEDV = left ventricular mass to volume ratio, WT= wall thickness, LVEF = left ventricular ejection fraction, LV GFI = left ventricular global function index, GLS = Global Longitudinal Strain, AoD = aortic distensibility of the descending aorta. P-values that were below the reporting threshold provided by R are shown as <1.00x10^-250^.

## Supplementary Table 5. Associations between hypertension and CMR metrics in fully adjusted models stratified by time since hypertension diagnosis

| **Metric** | **Five years or less** | **Six to 10 years** | **Eleven to 20 years** | **More than 20 years** |
| --- | --- | --- | --- | --- |
|  | **(n= 2,642)** | **(n= 2,653)** | **(n= 5,849)** | **(n= 3,963)** |
| LAVi | 0.128* [0.076, 0.181] | 0.125* [0.075, 0.175] | 0.151* [0.111, 0.190] | 0.231* [0.184, 0.278] |
|  | 1.59x10-6 | 8.51x10-7 | 5.68x10-14 | 6.31x10-22 |
| LAEF | -0.168* [-0.220, -0.116] | -0.075* [-0.125, -0.026] | -0.081* [-0.120, -0.042] | -0.171* [-0.218, -0.125] |
|  | 2.05x10-10 | 0.0027 | 4.40x10-5 | 6.48x10-13 |
| LVEDVi | 0.115* [0.072, 0.157] | 0.096* [0.054, 0.138] | 0.090* [0.057, 0.122] | 0.111* [0.073, 0.150] |
|  | 1.56x10-7 | 6.49x10-6 | 5.21x10-8 | 1.27x10-8 |
| LVSVi | 0.118* [0.073, 0.163] | 0.097* [0.053, 0.142] | 0.107* [0.072, 0.141] | 0.131* [0.091, 0.172] |
|  | 3.14x10-7 | 1.58x10-5 | 9.55x10-10 | 2.23x10-10 |
| LVMi | 0.251* [0.214, 0.288] | 0.229* [0.193, 0.265] | 0.244* [0.216, 0.272] | 0.314* [0.281, 0.347] |
|  | 2.79x10-40 | 2.68x10-35 | 1.64x10-65 | 9.04x10-77 |
| LVM/LVEDV | 0.171* [0.129, 0.212] | 0.164* [0.123, 0.205] | 0.198* [0.166, 0.229] | 0.260* [0.222, 0.297] |
|  | 8.74x10-16 | 2.86x10-15 | 6.33x10-35 | 2.22x10-42 |
| Maximal WT | 0.210* [0.172, 0.248] | 0.195* [0.158, 0.231] | 0.238* [0.210, 0.267] | 0.312* [0.277, 0.346] |
|  | 2.73x10-27 | 1.01x10-25 | 1.00x10-59 | 2.45x10-70 |
| LVEF | 0.017 [-0.029, 0.063] | 0.021 [-0.023, 0.066] | 0.048* [0.014, 0.083] | 0.054* [0.013, 0.095] |
|  | 0.4701 | 0.3469 | 0.0060 | 0.0092 |
| LV GFI | -0.058* [-0.102, -0.015] | -0.055* [-0.098, -0.013] | -0.049* [-0.081, -0.016] | -0.071* [-0.110, -0.033] |
|  | 0.0079 | 0.0098 | 0.0032 | 3.03x10-4 |
| GLS | 0.060* [0.009, 0.112] | 0.062* [0.013, 0.110] | 0.018 [-0.021, 0.056] | 0.079* [0.033, 0.125] |
|  | 0.0210 | 0.0136 | 0.3705 | 8.32x10-4 |
| Native T1 | -0.072* [-0.111, -0.033] | -0.051* [-0.090, -0.012] | -0.049* [-0.078, -0.020] | -0.072* [-0.107, -0.038] |
|  | 2.63x10-4 | 0.0098 | 8.65x10-4 | 3.39x10-5 |
| AoD | -0.159* [-0.196, -0.122] | -0.176* [-0.213, -0.140] | -0.171* [-0.199, -0.144] | -0.187* [-0.219, -0.155] |
|  | 2.62x10-17 | 5.90x10-21 | 2.66x10-34 | 1.06x10-29 |

**Supplementary Table 5 footnote.** Results are standardised Beta coefficients and 95% CIs with corresponding p-values, representing SD change in CMR metrics associated with hypertension status in participants stratified by the period of time since hypertension diagnosis. Each row is one model, reference group is no hypertension. Models include adjustment for age, sex, ethnicity, Townsend deprivation index, alcohol, BMI, smoking, diabetes, high cholesterol. T1 with additional adjustment is additionally adjusted by myocardial wall thickness, haematocrit and heart rate. An * indicates p-values that were significant after adjustment for multiple testing with a false discovery rate of 5%. CMR= cardiovascular magnetic resonance, LAVi = maximum left atrial volume indexed to body surface area, LAEF= left atrial ejection fraction, LVEDVi = left ventricular end-diastolic volume indexed to body surface area, LVSVi = left ventricular stroke volume indexed to body surface area, LVMi = left ventricular mass indexed to body surface area, LVM/LVEDV = left ventricular mass to volume ratio, WT= wall thickness, LVEF = left ventricular ejection fraction, LV GFI = left ventricular global function index, GLS = Global Longitudinal Strain, AoD = aortic distensibility of the descending aorta.

## Supplementary Table 6. Associations of treated hypertension with CMR metrics in fully adjusted linear regression models in men and women (compared with no hypertension)

|  | **Treated hypertension SBP below threshold** | | | **Treated hypertension SBP above threshold** | | |
| --- | --- | --- | --- | --- | --- | --- |
| **Metric** | **Whole sample** | **Women** | **Men** | **Whole sample** | **Women** | **Men** |
| LAVi | 0.082* | 0.023 | 0.159* | 0.288* | 0.301* | 0.274* |
|  | 0.0010 | 0.4602 | 6.49x10^-5^ | 1.57x10^-31^ | 2.20x10^-17^ | 4.86x10^-15^ |
| LAEF | -0.198* | -0.123* | -0.292* | -0.137* | -0.206* | -0.081* |
|  | 1.38x10^-16^ | 6.42x10^-5^ | 8.15x10^-15^ | 6.55x10^-9^ | 4.00x10^-9^ | 0.0131 |
| LVEDVi | 0.013 | -0.012 | 0.049 | 0.164* | 0.177* | 0.168* |
|  | 0.5307 | 0.6326 | 0.1683 | 3.82x10^-16^ | 2.28x10^-11^ | 3.48x10^-8^ |
| LVSVi | -0.015 | 0.005 | -0.034 | 0.230* | 0.217* | 0.250* |
|  | 0.5080 | 0.8506 | 0.3529 | 1.33x10^-26^ | 2.57x10^-13^ | 2.83x10^-15^ |
| LVMi | 0.028 | 0.032 | 0.038 | 0.376* | 0.376* | 0.384* |
|  | 0.1050 | 0.1192 | 0.2095 | 1.81x10^-105^ | 4.40x10^-63^ | 2.45x10^-48^ |
| LVM/LVEDV | 0.022 | 0.044 | 0.003 | 0.274* | 0.286* | 0.259* |
|  | 0.2659 | 0.0627 | 0.9334 | 1.45x10^-45^ | 1.71x10^-27^ | 4.91x10-20 |
| Maximal WT | 0.055* | 0.070* | 0.046 | 0.335* | 0.355* | 0.317* |
|  | 0.0018 | 7.96x10-4 | 0.1187 | 1.73x10-80 | 1.48x10-50 | 2.82x10-34 |
| LVEF | -0.034 | 0.034 | -0.117* | 0.138* | 0.090* | 0.169* |
|  | 0.1183 | 0.2250 | 7.02x10-4 | 7.69x10-11 | 0.0039 | 7.40x10-9 |
| LV GFI | -0.036 | 0.008 | -0.094* | -0.004 | -0.057 | 0.032 |
|  | 0.0894 | 0.7882 | 0.0030 | 0.8435 | 0.0689 | 0.2363 |
| GLS | 0.055* | 0.029 | 0.088* | 0.036 | 0.078 | 0.009 |
|  | 0.0258 | 0.3948 | 0.0161 | 0.1360 | 0.0382 | 0.7854 |
| Native T1 | 0.036 | -0.009 | 0.071* | -0.023 | -0.087* | 0.007 |
|  | 0.0702 | 0.7393 | 0.0122 | 0.2038 | 0.0017 | 0.7667 |
| AoD | 0.097* | 0.066* | 0.124* | -0.283* | -0.357* | -0.224* |
|  | 1.42x10-7 | 0.0118 | 1.15x10-6 | 2.72x10-59 | 5.86x10-39 | 3.41x10-24 |

**Supplementary Table 6 footnote.** Results are standardised beta coefficients and p-values, representing SD change in CMR metrics associated with hypertension diagnosis + hypertension treatment confirmed by Primary Care records compared with no hypertension. Fully adjusted models include adjustment for age, sex, ethnicity, Townsend deprivation index, alcohol, BMI, smoking, diabetes, high cholesterol. T1 with additional adjustment is additionally adjusted by myocardial wall thickness, haematocrit and heart rate. * indicates p-values that were significant after adjustment for multiple testing with a false discovery rate of 5%. CMR= cardiovascular magnetic resonance, LAVi = maximum left atrial volume indexed to body surface area, LAEF= left atrial ejection fraction, LVEDVi = left ventricular end-diastolic volume indexed to body surface area, LVSVi = left ventricular stroke volume indexed to body surface area, LVMi = left ventricular mass indexed to body surface area, LVM/LVEDV = left ventricular mass to volume ratio, WT= wall thickness, LVEF = left ventricular ejection fraction, LV GFI = left ventricular global function index, GLS = Global Longitudinal Strain, AoD = aortic distensibility of the descending aorta, SBP= systolic blood pressure.

## Supplementary Table 7. Associations of hypertension diagnosis with CMR metrics in fully adjusted linear regression models by ethnicity

|  | **White  N= [21,225 - 36,313]** | | | **Asian N = [222 - 392]** | | | **Black N = [128 - 241]** | | | **Chinese N = [70 - 108]** | | |
| --- | --- | --- | --- | --- | --- | --- | --- | --- | --- | --- | --- | --- |
| **Metric** | **Unadj** | **Age + sex** | **Fully adj** | **Unadj** | **Age + sex** | **Fully adj** | **Unadj** | **Age + sex** | **Fully adj** | **Unadj** | **Age + sex** | **Fully adj** |
| LAVi | 0.123* | 0.154* | 0.158* | 0.245 | 0.239 | 0.260 | 0.176 | 0.236 | 0.190 | 0.204 | 0.233 | 0.453 |
|  | 9.59x10-20 | 2.62x10-28 | 6.99x10-26 | 0.0315 | 0.0539 | 0.0481 | 0.2460 | 0.1346 | 0.2758 | 0.4102 | 0.3658 | 0.1404 |
| LAEF | -0.214* | -0.152* | -0.115* | -0.329* | -0.201 | -0.234 | -0.096 | -0.069 | -0.050 | -0.064 | -0.059 | -0.324 |
|  | 1.46x10-56 | 3.25x10-28 | 9.59x10-15 | 0.0066 | 0.1187 | 0.0869 | 0.4649 | 0.6164 | 0.7358 | 0.8250 | 0.8442 | 0.3615 |
| LVEDVi | -0.000 | -0.020 | 0.101* | 0.108 | 0.147 | 0.155 | 0.052 | 0.180 | 0.182 | -0.028 | -0.061 | 0.027 |
|  | 0.9951 | 0.0804 | 1.75x10-16 | 0.2806 | 0.1586 | 0.1576 | 0.7242 | 0.2024 | 0.2361 | 0.8791 | 0.7307 | 0.8972 |
| LVSVi | -0.022 | -0.009 | 0.114* | 0.138 | 0.199 | 0.202 | -0.099 | 0.014 | -0.008 | -0.159 | -0.203 | -0.214 |
|  | 0.0785 | 0.4706 | 1.68x10-18 | 0.1787 | 0.0732 | 0.0860 | 0.5007 | 0.9261 | 0.9581 | 0.4446 | 0.3511 | 0.4102 |
| LVMi | 0.373* | 0.255* | 0.257* | 0.510* | 0.421* | 0.337* | 0.376* | 0.428* | 0.363* | 0.479 | 0.393 | 0.288 |
|  | 8.69x10-206 | 7.54x10-146 | 1.83x10-128 | 4.42x10-7 | 1.78x10-5 | 7.91x10-4 | 0.0108 | 0.0010 | 0.0090 | 0.0358 | 0.0678 | 0.2627 |
| LVM/LVEDV | 0.470* | 0.343* | 0.197* | 0.576* | 0.370* | 0.242 | 0.396* | 0.292 | 0.201 | 0.642* | 0.570* | 0.285 |
|  | < 1.00x10^-250^ | 1.78x10-193 | 3.88x10-61 | 3.44x10-6 | 0.0041 | 0.0638 | 0.0158 | 0.0658 | 0.2339 | 0.0060 | 0.0176 | 0.3126 |
| Maximal WT | 0.552* | 0.390* | 0.238* | 0.646* | 0.445* | 0.347* | 0.328 | 0.338* | 0.204 | 0.774* | 0.682* | 0.693* |
|  | < 1.00x10^-250^ | 5.65x10-276 | 1.95x10-105 | 2.27x10-7 | 1.38x10-4 | 0.0030 | 0.0465 | 0.0268 | 0.1626 | 0.0079 | 0.0128 | 0.0262 |
| LVEF | -0.014 | 0.036* | 0.040* | 0.057 | 0.090 | 0.078 | -0.220 | -0.238 | -0.274 | -0.166 | -0.152 | -0.336 |
|  | 0.2459 | 0.0029 | 0.0023 | 0.6173 | 0.4546 | 0.5389 | 0.1485 | 0.1261 | 0.1084 | 0.4833 | 0.5164 | 0.2313 |
| LV GFI | -0.228* | -0.124* | -0.055* | -0.186 | -0.064 | -0.013 | -0.365* | -0.340* | -0.345* | -0.470 | -0.422 | -0.453 |
|  | 9.65x10-78 | 5.41x10-27 | 9.97x10-6 | 0.0873 | 0.5644 | 0.9117 | 0.0093 | 0.0150 | 0.0236 | 0.0537 | 0.0757 | 0.1160 |
| GLS | 0.073* | 0.020 | 0.044* | 0.275 | 0.283 | 0.306* | -0.032 | 0.006 | 0.015 | 0.289 | 0.438 | 0.504 |
|  | 1.09x10-7 | 0.1513 | 0.0025 | 0.0314 | 0.0349 | 0.0299 | 0.8272 | 0.9687 | 0.9242 | 0.3190 | 0.1200 | 0.1299 |
| Native T1 | -0.162* | -0.091* | -0.062* | -0.002 | 0.116 | 0.155 | 0.188 | 0.104 | 0.001 | 0.133 | 0.286 | 0.282 |
|  | 2.01x10-51 | 1.98x10-18 | 2.15x10-8 | 0.9853 | 0.3033 | 0.1952 | 0.1517 | 0.4088 | 0.9960 | 0.5478 | 0.1818 | 0.2558 |
| AoD | -0.379* | -0.176* | -0.173* | -0.583* | -0.271* | -0.191 | -0.332* | -0.145 | -0.119 | -0.692* | -0.452 | -0.406 |
|  | 7.55x10-249 | 3.95x10-71 | 8.59x10-60 | 3.12x10-9 | 0.0016 | 0.0355 | 0.0076 | 0.2064 | 0.3287 | 0.0034 | 0.0438 | 0.1166 |

**Supplementary Table 7 footnote**. Results are standardised Beta coefficients and p-values, representing SD change in CMR metrics associated with diagnosed hypertension status, stratified by ethnic group. The reference group is no hypertension within each ethnic group. Models include adjustment for age, sex, Townsend deprivation index, alcohol, BMI, smoking, diabetes, high cholesterol. T1 with additional adjustment is additionally adjusted by myocardial wall thickness, haematocrit and heart rate. An * indicates p-values that were significant after adjustment for multiple testing with a false discovery rate of 5%. CMR= cardiovascular magnetic resonance, LAVi = maximum left atrial volume indexed to body surface area, LAEF= left atrial ejection fraction, LVEDVi = left ventricular end-diastolic volume indexed to body surface area, LVSVi = left ventricular stroke volume indexed to body surface area, LVMi = left ventricular mass indexed to body surface area, LVM/LVEDV = left ventricular mass to volume ratio, WT= wall thickness, LVEF = left ventricular ejection fraction, LV GFI = left ventricular global function index, GLS = Global Longitudinal Strain, AoD = aortic distensibility of the descending aorta. P-values that were below the reporting threshold provided by R are shown as <1.00x10^-250^.
